# Evaluation of flow cytometry for the detection of bacteria in biological fluids

**DOI:** 10.1371/journal.pone.0220307

**Published:** 2019-08-07

**Authors:** Elisa Rubio, Yuliya Zboromyrska, Jordi Bosch, Mariana J. Fernandez-Pittol, Berta I. Fidalgo, Assumpta Fasanella, Anna Mons, Angely Román, Climent Casals-Pascual, Jordi Vila

**Affiliations:** 1 Department of Microbiology, Biomedical Diagnostic Center (BDC), Hospital Clinic, University of Barcelona, Barcelona, Spain; 2 Consorci del Laboratori Intercomarcal de l´Alt Penedès, l´Anoia i el Garraf, Vilafranca del Penedès, Barcelona, Spain; 3 ISGlobal, Barcelona, Institute for Global Health, Barcelona, Spain; Xavier Bichat Medical School, INSERM-CNRS - Université Paris Diderot, FRANCE

## Abstract

**Objectives:**

Conventional microbiological procedures for the isolation of bacteria from biological fluids consist of culture on solid media and enrichment broth. However, these methods can delay the microbiological identification for up to 4 days. The aim of this study was to evaluate the analytical performance of Sysmex UF500i (Sysmex, Kobe, Japan) as a screening method for the detection of bacteria in different biological fluids in comparison with direct Gram staining and the conventional culture on solid media and enrichment broth.

**Methods:**

A total of 479 biological fluid samples were included in the study (180 ascitic, 131 amniotic, 56 synovial, 40 cerebrospinal, 36 pleural, 24 peritoneal, 9 bile and 3 pericardial fluids). All samples were processed by conventional culture methods and analyzed by flow cytometry. Direct Gram staining was performed in 339 samples. The amount of growth on culture was recorded for positive samples.

**Results:**

Bacterial and white blood cell count by flow cytometry was significantly higher among culture positive samples and samples with a positive direct Gram stain compared to culture negative samples. Bacterial count directly correlated with the amount of growth on culture (Kruskall-Wallis H χ^2^(3) = 11.577, p = 0.009). The best specificity (95%) for bacterial count to predict culture positivity was achieved applying a cut-off value of 240 bacteria/μL.

**Conclusions:**

Bacterial and white blood cell counts obtained with flow cytometry correlate with culture results in biological fluids. Bacterial count can be used as a complementary method along with the direct Gram stain to promptly detect positive samples and perform other diagnostic techniques in order to accelerate the bacterial detection and identification.

## Introduction

Early detection and identification of bacteria causing infection in biological fluids such as cerebrospinal, abdominal (ascitic, peritoneal and bile), synovial, pleural and amniotic fluids, is crucial for prompt and adequate antibiotic treatment and correct management of infection [1–3]. The classical microbiological methods to isolate bacteria from biological fluids consist of culture on solid media and enrichment broth. However, the detection and identification of bacteria following these methods can be delayed between 18 hours and 4 days. Direct Gram staining provides rapid identification information but it has a low diagnostic value since it requires a high bacterial inoculum to be positive [4–6]. The collection of biological fluids in blood culture bottles was introduced 20 years ago, increasing bacterial recovery and sometimes reducing the time prior to identification [7]. Molecular techniques based on multiplex polymerase chain reactions (PCRs) are a good and rapid alternative for the detection of bacterial genes in biological fluid. However, they also have the disadvantage of being expensive and aimed at specific pathogens, thereby detecting only a limited number of microorganisms [8].

Flow cytometry could improve time to microbiological diagnosis by directly detecting bacteria and other cells in different biological fluids [9]. The Sysmex UF500i (Sysmex, Kobe, Japan) is a fluorescence flow cytometer intended for urinalysis. It is able to classify and quantify urine cells, including red blood cells (RBC), white blood cells (WBC) and epithelial cells (EC). This instrument has two chambers:one chamber is intended for the detection of all other particles, where the nucleic-acid containing cells are stained with polymethine dye; the other chamber is intended for the detection of bacteria where only nucleic acids in bacteria are stained. After staining, the samples are transported to a flow cell where they are analyzed by a red semiconductor laser. Particle characterization is based on forward-scatter light, side-scatter light and fluorescence. Currently, flow cytometry has been used in clinical practice to detect culture-negative urine samples based on bacterial and WBC counts [10,11]. However, it has also been increasingly used as a screening method to rapidly identify positive urine samples with high bacterial concentration to perform direct identification and antimicrobial susceptibility testing [12–14].

The aim of this study was to evaluate the analytical performance of Sysmex UF-500i as a screening method for the detection of bacteria in different biological fluids in comparison with direct Gram staining and the conventional culture on solid media and enrichment broth.

## Materials and methods

### Sample inclusion

Consecutive biological fluid samples received to the Clinical Microbiology Department of a 700-bed university hospital, during working days in the morning period (8 a.m– 5 p.m) between June 2014 and November 2016 were included in the study. The samples were collected in sterile tubes with no chemical preservatives and processed immediately after arrival.

Samples were processed by the routine microbiological methods and subsequently analyzed by the Sysmex UF-500i (Sysmex, Kobe, Japan). The specimens with insufficient volume (less than 1 mL) or presenting excess viscosity to be processed by flow cytometry were excluded (data not recorded).

### Routine microbiological procedures

Gram stain screening was performed in samples classified as urgent by the attending clinician. Biological fluids were seeded manually on blood agar (Oxoid, Madrid, Spain), chocolate agar (Becton Dickinson, Heidelberg, Germany), MacConkey agar (Becton Dickinson) and/or Schaedler agar (Becton Dickinson), and thioglycollate broth (Becton Dickinson) according to usual protocols [15]. The positive samples were classified according to the bacterial growth observed on the plates: growth only in the thioglycollate broth subcultures, <10 grown colonies, 10–100 grown colonies and >100 grown colonies on the first seeded plates. The final bacterial identification was achieved using MALDI-TOF MS (matrix-assisted laser desorption/ionization time-of-flight mass spectrometry) (Bruker, Bremen, Germany). Detection of *Mycoplasma hominis* and *Ureaplasma* spp., in amniotic fluids only, was performed using the Mycoplasma IST2 (Biomérieux, Marcy l’Étoile, France).

### Flow cytometry

All samples were analyzed by flow cytometry using the UF-500i and following manufacturer’s recommendations immediately after culture inoculation. Samples were analyzed manually; flow cytometry required 1 mL of each sample. In this study, only bacterial, and WBC counts provided by the UF-500i were used for sample interpretation. A high and a low positive control were processed daily as recommended by the manufacturer’s instructions.

### Statistical analysis

Statistical analysis was performed using IBM SPSS Statistics 22.0 (Armonk, New York). Clinical and microbiological data were obtained from medical records in a fully anonymized and de-identified manner and entered in a password-protected database. Only the authors had access to identifying information. The different parameters measured by flow cytometry were defined as dependent variables using the culture and Gram staining results as independent variables. The distribution of independent variables across groups were compared using the Mann-Whitney U test for the comparison of two independent variables, and the Kruskall-Wallis H test for the comparison of more than two independent variables. The differences were considered statistically significant with a *p*-value <0.05.

The receiver operating characteristic (ROC) curves were used to compare the UF-500i bacterial and WBC counts to the culture results obtained by routine procedure. The cut-off values were chosen based on best balance between sensitivity and specificity, giving priority to specificity in order to detect culture positive samples with IBM SPSS Statistics 22.0 (Armonk, New York).

### Ethical statement

The study was reviewed by the Ethical Research Committee at Hospital Clínic of Barcelona, Spain, waiving the requirements for approval and consent. The study was carried out without additional intervention of patients, all samples were processed following the routine procedures, and patient’s data were anonymized before the analysis. Informed consent was not required.

## Results

### Gram staining and culture results

A total of 493 biological fluids were analyzed by flow cytometry but 14 samples positive only for *Candida* spp. were excluded. Finally, 479 biological fluids originating from 408 patients were included in the study. The mean age was 57 years (interquartile range (IQR): 38–71) and 58.9% were female. The samples included as well as the number of positive and negative cultures and the final bacterial identification for each type of biological fluid are shown in Table 1. In 83 (17.3%) samples bacterial growth was detected by culture.

**Table 1:** Microbiological culture results and bacterial identifications obtained from the biological fluids included in the study.

Culture (N)	Ascitic fluid (180)	Amniotic fluid (131)	Synovial fluid (56)	Cerebrospinal fluid (40)	Peritoneal fluid (24)	Bile (9)	Pleural fluid (36)	Pericardial fluid (3)
Negative, n (%)	162 (90)	107 (81,7)	45 (80,4)	27 (67,5)	14 (58,3)	3 (33,3)	35 (97,2)	3 (100)
Positive, n (%)	18 (10)	24 (18,3)	11 (19,6)	13 (32,5)	10 (41,7)	6 (66,7)	1 (2,8)	0 (0)
Polymicrobial, n (%)	4^c^ (22,2)	6^d^ (25)	0 (0)	3^e^ (23,1)	4^f^ (40)	5^g^ (83,3)	0 (0)	0
Monomicrobial culture, n (%)	14 (77,8)	18 (75)	11 (100)	10 (76,9)	6 (60)	1 (16,7)	1 (100)	0
*Staphylococcus epidermidis* (10)	1	2	2	4	1	-	-	-
*Staphylococcus aureus* (8)	1	1	5	-	1	-	-	-
Other CNS^a^ **(**6)	3	-	1	2	0	-	-	-
Non-fermenter GNB^b^ (5)	1	1	1	-	2	-	-	-
*Enterobacteriaceae* (10)	2	3	1	2	1	1		
Anaerobe GNB ^b^ (4)	-	4	-	-	-	-	-	-
*Enterococcus* spp. (7)	5	-	-	2	-	-	-	-
*Streptococcus viridans* group (4)	1	1	-	-	1	-	1	-
*Streptococcus agalactiae* (3)	-	2	1	-	-	-	-	-
*Peptostreptococcus* spp.(1)	-	1	-	-	-	-	-	-
*Gardnerella vaginalis* (1)	-	1	-	-	-	-	-	-
*Mycoplasma hominis/* *Ureaplasma* spp. (2)	-	2	-	-	-	-	-	-

^a^ CNS: Coagulase negative staphylococci

^b^ GNB: Gram negative bacilli

^c^ *Pseudomonas aeruginosa* + *Bacteroides vulgatus*, *P. aeruginosa* + *Enterococcus faecium*, *Enterococcus faecalis* + CNS, *Streptococcus anginosus* +*E.faecalis*

^d^ *Mycoplasma hominis* + *Ureaplasma urealyticum* (2), *Streptococcus agalactiae* + *Candida albicans*, *S. agalactiae* + *Prevotella bivia*, *Fusobacterium* spp. + *C. albicans*, *Peptoniphilus harei* + *P. bivia*

^e^ *P. aeruginosa* + *Staphylococcus aureus*, *Clostridium perfingens*+ *Clostridium baratii*, *Enterobacter aerogenes* + *Klebsiella oxytoca* + *E. faecalis*;

^f^ Mixed flora not identified (2), *E*. *faecium + K*. *oxytoca*, *Citrobacter freundii* + *Klebsiella pneumoniae*

^g^ *P. aeruginosa* + *K. pneumoniae*, *Escherichia coli* + *Morganella morganii* + *K. oxytoca*, *Enterobacter aerogenes* + *P. aeruginosa*, *E. coli* + *Candida glabrata*, *E*, *faecium* + *P. aeruginosa*.

**Fig 1:**
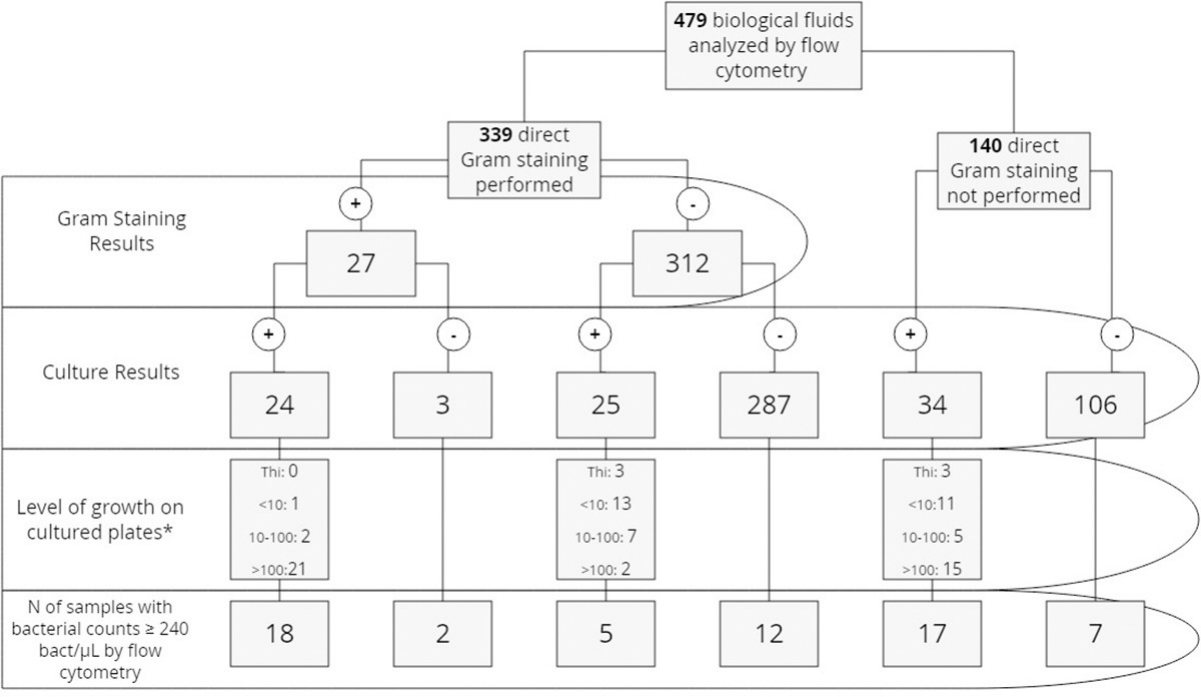
Gram staining, culture, level of growth on cultured plates and flow cytometry results of the biological samples included in the study. * Thi: growth only in the thioglycollate broth subcultures, <10 grown colonies on cultured plates, 10–100 grown colonies on cultured plates, >100 grown colonies on cultured plates.

Fig 1 summarizes the results obtained from the biological fluids included in the study according to culture, Gram staining, level of growth on cultured plates and flow cytometry results. Gram stain was requested in 339 (70.8%) samples presenting a sensitivity of 48.84% and a specificity of 98.97% for the total of samples in our study.

### Flow cytometry counts according to culture and Gram stain results

**Fig 2:**
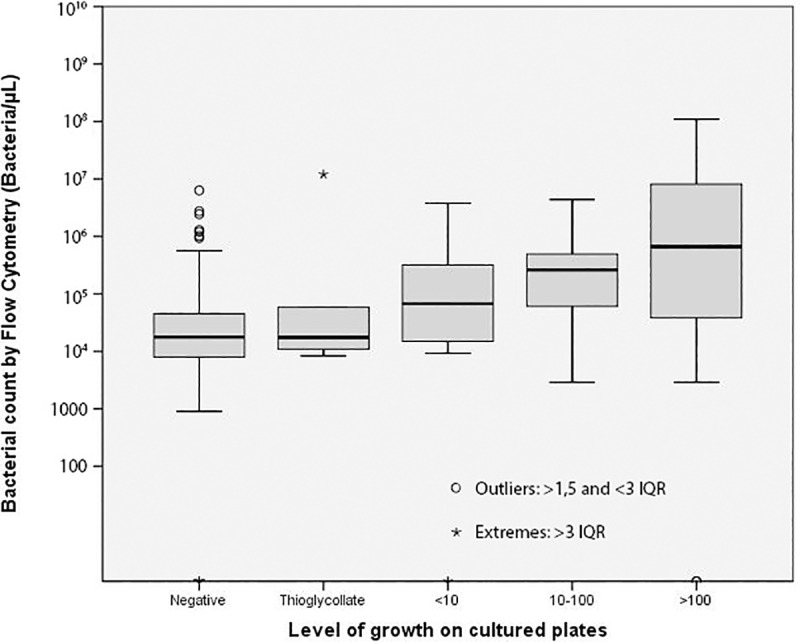
Distribution of bacterial count by flow cytometry according to the level of growth on cultured plates. IQR: interquartile range Thioglycollate: growth only in the thioglycollate broth subcultures, <10 grown colonies on cultured plates, 10–100 grown colonies on cultured plates, >100 grown colonies on cultured plates.

Table 2 shows the median and IQR values of WBC/μL and bacteria/μL count estimated by flow cytometry of the samples included in the study according to culture positivity, Gram stain and amount of growth on the plates. Fig 2 shows the distribution of bacterial count by flow cytometry according to the amount of growth on cultured plates.

**Table 2:** Median and interquartile ranges of flow cytometry parameters according to culture positivity direct gram staining and level of growth on cultured plates.

	Bacteria/μL			WBC/μL		
	Median	IQR		Median	IQR	
Culture negative (n = 396)	17.8	8–45.6	p<0.0001^a^	89.9	31.1–305.4	p = 0.009^a^
Culture positive (n = 83)	205.3	20.9–1178.4		291.5	27.2–3345.5	
Direct negative Gram staining (n = 312)	20.05	9.0–49	p<0.0001^a^	81.4	30.3–284.8	p = 0.008^a^
Direct positive Gram staining (n = 27)	1178.4	80–6792.8		384.6	37.1–3282.4	
Growth only in thioglycollate broth (n = 6)	17.5	10.3–3049.2	p = 0.009^b^	41.2	11.8–718.5	p = 0.31
Growth <10 colonies /plate (n = 25)	67.7	14.5–336.6		215.4	31.4–1515.5	
Growth 10–100 colonies /plate (n = 14)	261.4	56.9–586.9		2609.9	49.9–10562.9	
Growth >100 colonies /plate (n = 38)	671.9	37.0–8557.6		364.0	20.9–2404.9	

^a^ Mann-Whitney

^b^ Kruskall-Wallis H χ^2^(3) = 11.577

### Receiver operating characteristic (ROC) curves

**Fig 3:**
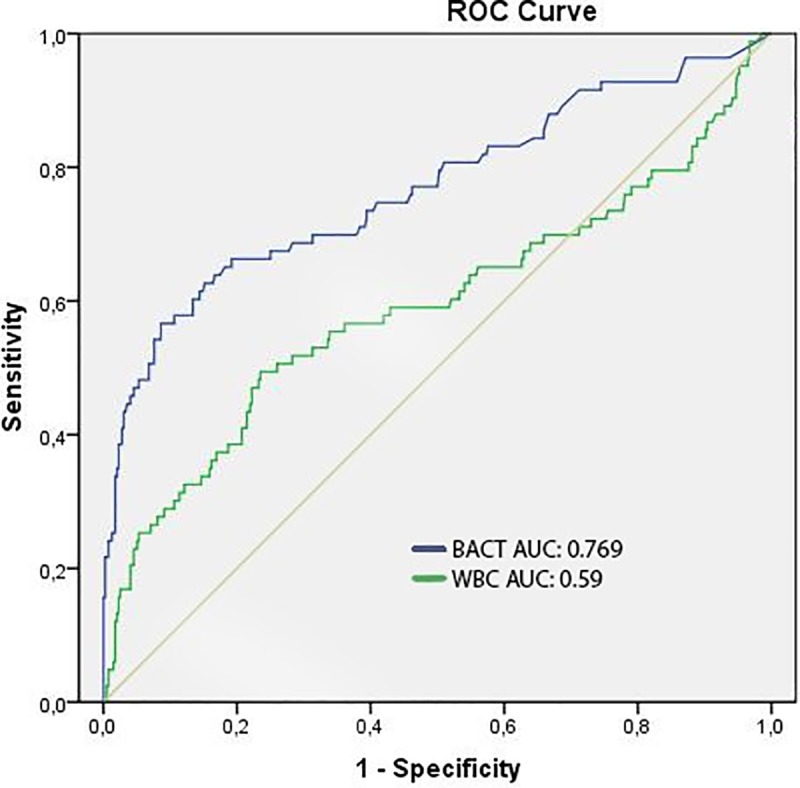
ROC curve for bacterial count (BACT) and leukocyte count (WBC) by flow cytometry *versus* culture positivity for 479 biological fluid samples with 83 samples positive by culture. AUC: Area under the curve.

The ROC curves of bacteria/μL and culture positivity yielded an area under the curve (AUC) of 0.769 (95%CI 0.703–0.834) for all samples (Fig 3) and an AUC of 0.879, 0.784, 0.785, 0.737 and 0.571 for amniotic, synovial, cerebrospinal, ascites and peritoneal fluids, respectively (S1 Fig). The ROC curves for pericardial, bile and pleural fluids were not calculated due to the low number of pericardial and bile fluids included and because of the low number of positive samples among pleural fluids (Table 1).

The ROC curve of WBC/μL and culture positivity yielded an AUC of 0.59 (95% CI 0.513–0.671) for the total of samples (Fig 3). The WBC counts were not used in further analysis since the data provided by this parameter alone or in combination with bacterial counts was not strong to predict culture positivity.

The optimal cut-off point from ROC analysis of bacterial count to predict culture positivity was established at 240 bacteria/μL, with a subsequent sensitivity of 48.2%, specificity of 94.7%, negative predictive value (NPV) of 89.7% and positive predictive value (PPV) of 65.6% for the total of biological fluids. Applying this cut-off value a 93% correlation between Gram stain and flow cytometry was obtained (Fig 1).

Amniotic fluids were analyzed separately due to the good performance of bacterial counts to predict culture positivity in this type of sample; here, the optimal cut-off point was established at 150 bacteria/μL with a sensitivity of 83.3%, specificity of 92.5%, NPV of 96.1% and PPV of 71.4%.

Applying the calculated cut-off point (240 bacteria/ μL) to the total of samples, 21 samples of 479(4,38%) with bacterial counts over the cut-off value by flow cytometry and culture negative were obtained from 20 patients (6 ascitic, 6 amniotic, 5 articular, 2 pericardial and 2 peritoneal fluids).

In 14 of these 20 patients (70%) a final diagnosis of infection was established based on clinical symptoms. Among these 14 patients, the infectious etiology was confirmed in 8 patients by other samples (such as blood cultures or other biological fluids) positive for bacterial culture in the same episode (two samples with positive direct Gram staining). The remaining 6 patients did not have any positive microbiological sample.

Twelve patients out of 20 (60%) with samples showing high bacterial counts by flow cytometry and culture negative results were under antibiotic treatment when the biological fluid was obtained.

## Discussion

Rapid bacterial detection in biological fluids is crucial for targeted antibiotic administration and management of the infection. Classical microbiological methods consist of culture on solid media and enrichment broth or collection of the samples in blood culture bottles. These methods can delay the microbiological results for up to 4 days. Direct detection and quantification of bacteria and WBC by flow cytometry allows rapid detection of presumed positive samples. We evaluated the flow cytometer UF-500i (Sysmex) as a screening method for the rapid detection of bacteria in different biological fluid samples and compared it with the growth on solid media and enrichment broth and with the results obtained by direct Gram stain. There was consensus between bacterial count by flow cytometer and culture results. The samples with a positive culture showed statistically higher bacterial counts than samples with negative culture, and the bacterial count directly correlated with the growth amount on the seeded plates (Table 2, Fig 2). Regarding Gram staining, samples with a positive direct Gram stain showed statistically higher bacterial counts than samples with negative Gram stain. Additionally, WBC count in culture positive samples was statistically higher compared to culture negative samples and in samples with positive direct Gram stain compared to samples with negative Gram stain, nevertheless WBC count do not improve the detection of positive samples comparing with bacterial count alone.

Direct detection and quantification of bacteria in sterile biological fluids by flow cytometry could accelerate the microbiological etiology detection. The main limitation is the low volume provided in many cases (less than 1 mL), not sufficient to perform flow cytometry analysis, and the excessive viscosity due to high human cell concentrations in some samples, which could impair the count of particles. Flow cytometry has been demonstrated to correctly classify and quantify WBC in cerebrospinal and peritoneal fluids [16–20]. Our results are in agreement with previous studies which have mainly evaluated bacterial counts by flow cytometry in cerebrospinal fluids, showing a good correlation with bacterial culture. However, these studies included a low number of bacterial meningitis cases (a maximum of 7% culture positive samples) [16,18,20]. Saito *et*. *al*. evaluated an experimental bacterial counter based on flow cytometry on different biological fluids. Applying a cut-off between 40–100 bacteria/μL according to the type of sample a 84.4% sensitivity and 86.0% specificity was obtained to predict culture positivity [21].

Samples with bacterial counts above the cut-off values show a high probability of being culture positive (65.6% PPV), thus warranting further diagnostic methods to accelerate microbiological identification. Many protocols based on screening by flow cytometry have been proposed for urine samples. Direct identification by mass-spectrometry, [12–14] direct antimicrobial susceptibility testing [13] and direct detection of extended spectrum β-lactamase and carbapenemase production [22] have been successfully performed for urine samples with high microbial concentration. Other studies proposed a short incubation step before direct identification for samples with lower bacterial concentration [23].

Although samples with low bacterial counts show low probability of being culture positive (89.7% NPV) microbiological culture should not be eliminated for biological fluids, as it has been suggested for urine cultures [10,11,24–29], due to the severity of these infections and the presence of false negatives in the screening performance.

Flow cytometry can barely replace Gram staining as a screening method in biological fluids. However, it can provide additional information regarding bacterial counts which along with the Gram staining results can be used for the decision criteria on whether to apply or not rapid identification or antibiotic susceptibility techniques. Moreover, in laboratories where Gram stain is not always performed due to high amount of samples, flow cytometry could be used as a screening method. Compared to Gram staining, flow cytometry has the advantages of being faster and easier to perform and a good correlation between both techniques (93%) with the same sensitivity (48–49%) was shown.

Although an initial inversion is required for the incorporation of flow cytometry to the microbiology laboratory, its use in biological fluids would provide a new application, especially for laboratories already performing urine screening with flow cytometry.

The main limitations of our study were that some biological fluids (data not recorded) were not adequate for flow cytometry analysis: some cerebrospinal fluids were excluded since the volume provided was under 1 mL; other samples like some synovial and abdominal fluids could not be analyzed by flow cytometry due to excess of viscosity.

We analyzed the samples together without taking into account particular characteristics of the different types of fluid which could influence in the performance of the assay. Finally, further diagnostic techniques such as direct 16s rRNA gene sequencing were not performed on culture negative samples with high bacterial counts by flow cytometry. However, in 14/20 patients with samples presenting high bacterial counts by flow cytometry and culture negative results, a final diagnosis of infection was established, and 12/20 patients were under antibiotic treatment when the sample was obtained. These results suggest that bacterial counts detected by flow cytometry may have corresponded to viable but nonculturable bacterial cells [30].

To our knowledge, this is the firsts study to evaluate the UF-500i performance for bacterial detection in a variety of biological samples.

In conclusion, the bacterial and WBC load according to the UF-500i correlate with culture results in biological fluids and can be used as a complementary method along with the direct Gram stain to promptly detect positive samples and perform other diagnostic techniques in order to accelerate the bacterial detection and identification.

## Supporting information

S1 Fig ROC curves for bacterial counts by flow cytometry *versus* culture positivity for each analyzed biological fluid. AUC: Area under the curve.
(TIF)
