# Supplementary figures and images for: Evaluation of flow cytometry for the detection of bacteria in biological fluids
Source: PLoS One. 2019 Aug 7;14(8):e0220307. doi: 10.1371/journal.pone.0220307 (PMC6685611; doi:10.1371/journal.pone.0220307)

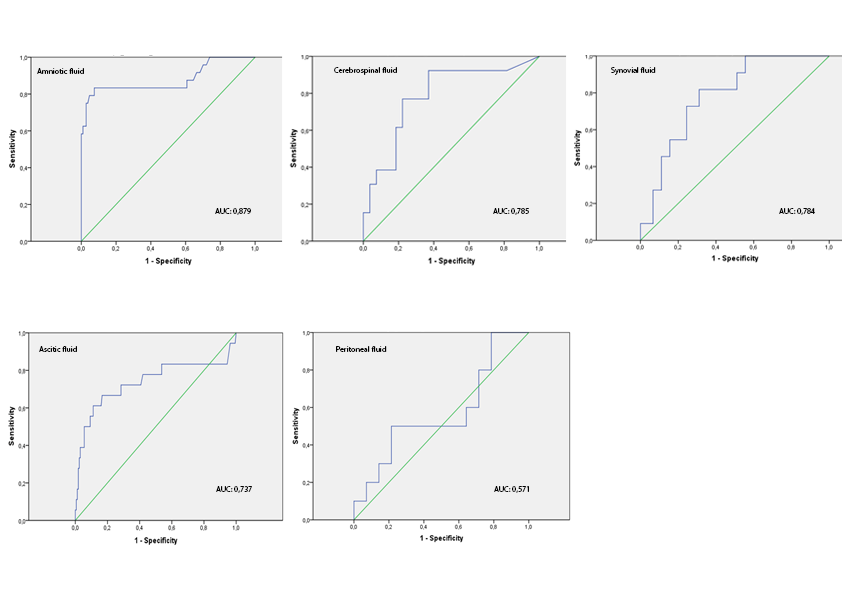

Supplement: S1 Fig — AUC: Area under the curve. (TIF) [file pone.0220307.s001.tif]
